# Acute Indigestion in the Cow1Presented before the Keystone Veterinary Medical Association, October, 1897.

**Published:** 1897-12

**Authors:** W. H. Ridge

**Affiliations:** Trevose, Pa.


### ACUTE INDIGESTION IN THE COW.[1]

(*Synonyms:* Plenalvia; Grain-sick; Maw-bound.)

By W. H. Ridge, V.M.D.,
TREVOSE, PA.

Indigestion in the cow takes many forms, owing to the large size and divisions of the stomach, which need no description here. It is not our intention to consider indigestion in all of its phases, but will confine ourselves to one—the engorgement of the rumen with rich or soft food.

With all care, many cows die from eating too much feed; getting loose, they gain access to the feed-bin, eating a great quantity of meal—sometimes as much as two bushels; or escape into a corn-field, or an orchard, or eat large quantities of potatoes, cabbage-roots, etc. This trouble has been described by our oldest writers, and we do not expect to give anything new, but to bring the subject up for discussion.

Of course, the cause is a voracious appetite, together with a stomach so large as to be capable of holding an immense quantity of food, and the opportunity of filling it.

I know of no disease in the cow in which the symptoms vary more than in this complaint, the result depending on the amount of food ingested and the power of digesting large amounts, the physical condition, and the care of the animal after engorgement. Sometimes the symptoms are very acute, and appear soon after eating; others not for, perhaps, two or three days. If the food be of easily fermenting character, the symptoms develop much sooner. In those cases that develop soon, tympanites is the chief trouble, with its train of symptoms—neck extended, as if trying to vomit, very uneasy, bellowing, and becoming frantic. If badly swollen, these cases soon die from asphyxia if not relieved.

[1] Presented before the Keystone Veterinary Medical Association, October, 1897.

Another animal may show no signs of sickness for twenty-four to thirty hours after eating a large quantity of rich food, except, perhaps, a fulness of the abdomen. During this time, if closely watched, it will be noticed that the animal is sluggish and not eating as freely as the remainder of the herd. The first thing the owner will notice is the loss of milk—the animal milking well at night, and found dry in the morning, with a profuse diarrhœa. In a few hours it gets down, and is unable to rise, making one think it paralyzed. Or, perhaps, the bowels are torpid and have no evacuation for the first day, the animal grunting when moved; nose dry, with suppression of milk, as in the other phase of the trouble.

The respiration may lead you to think you have a case of pneumonia, or of chest-trouble; but a careful examination of these parts will exclude these. The rumen has a doughy feel, and pits on pressure, and is seldom, in this stage, tympanitic. Rumination is suspended; the appetite lost; the body becomes cold, and the pulse feeble and frequent; the breath often fetid. When fermentation is present you will get a tympanitic feel over a doughy mass. The animal gets down, mostly on the right side; eyes turned back, and dies in spasms.

These cases are not so difficult to diagnose as when we have diarrhœa, suppression of milk, and down and unable to rise, with no history. I have seen several mistakes made in this one condition.

The diagnosis is to be made from paralysis œstrum, eating frosted food, parturient apoplexy, pneumonia, choking, tubercular diarrhœa, impaction of the third stomach or omasum, poisons, volvulus.

The termination is, of course, according to the amount ingested and the manner of treatment. Most animals recover, but lose the milk until the next calving.

The lesions are those of inflammation of the bowels and asphyxia, and sometimes rupture of the bowels, with a large amount of food, the lesion depending upon the length of time before death.

When first in practice I was led to believe that the animal should have a purgative; but after trying this mode of treatment for a while, and many, too many, dying, I began trying to find other treatment which would give me better results. I well remember a stable of cows that twenty-four hours before got into an orchard and ate as many apples as they wished; three-fourths of the animals were down, purging, and unable to rise, groaning and at times struggling. It happened at night, and I had only a colic-mixture with me. Not having any choice, I dosed each with it, when they all recovered without the trouble I experienced before. I have watched my cases since, and am decidedly opposed to giving physics to commence with. I allow frequently small quantities of water, and give internally a fever-mixture with nux vomica. One that I have used with success is: ℞.—Tr. aconiti, ʒj; tr. belladonn. ʒij; sulph. cinchonæ, ʒij; tr. nux vom. ʒiv; spt. nitr. dulc. q. s. ℥ij. Sig. Teaspoonful every hour or two hours, as case needs. As soon as the diarrhœa commences, and the animal is in pain, or as soon as pain manifests itself, give ℞—Ether. sulph. 6–48; tr. asafœtida, 6–48; spt. camph. 3–24; ext. cannabis Ind. fl., 3–24; ol. pep. menth. $\frac{1}{8}$–1. Sig. Tablespoonful doses, diluted; or alternate these remedies, and the purgative will be rarely needed in this condition. Of course we are not to mistake an impaction of dry material, which will need a brisk purgative, and we will consider these cases at another meeting.

